# How State Taxes and Policies Targeting Soda Consumption Modify the Association between School Vending Machines and Student Dietary Behaviors: A Cross-Sectional Analysis

**DOI:** 10.1371/journal.pone.0098249

**Published:** 2014-08-01

**Authors:** Daniel R. Taber, Jamie F. Chriqui, Renee Vuillaume, Frank J. Chaloupka

**Affiliations:** 1 Institute for Health Research and Policy, University of Illinois at Chicago, Chicago, Illinois, United States of America; 2 John F. Kennedy School of Government, Harvard University, Cambridge, Massachusetts, United States of America; 3 Department of Economics, University of Illinois at Chicago, Chicago, Illinois, United States of America; Indiana University, United States of America

## Abstract

**Background:**

Sodas are widely sold in vending machines and other school venues in the United States, particularly in high school. Research suggests that policy changes have reduced soda access, but the impact of reduced access on consumption is unclear. This study was designed to identify student, environmental, or policy characteristics that modify the associations between school vending machines and student dietary behaviors.

**Methods:**

Data on school vending machine access and student diet were obtained as part of the National Youth Physical Activity and Nutrition Study (NYPANS) and linked to state-level data on soda taxes, restaurant taxes, and state laws governing the sale of soda in schools. Regression models were used to: 1) estimate associations between vending machine access and soda consumption, fast food consumption, and lunch source, and 2) determine if associations were modified by state soda taxes, restaurant taxes, laws banning in-school soda sales, or student characteristics (race/ethnicity, sex, home food access, weight loss behaviors.)

**Results:**

Contrary to the hypothesis, students tended to consume 0.53 fewer servings of soda/week (95% CI: -1.17, 0.11) and consume fast food on 0.24 fewer days/week (95% CI: -0.44, -0.05) if they had in-school access to vending machines. They were also less likely to consume soda daily (23.9% vs. 27.9%, average difference = -4.02, 95% CI: -7.28, -0.76). However, these inverse associations were observed primarily among states with lower soda and restaurant tax rates (relative to general food tax rates) and states that did not ban in-school soda sales. Associations did not vary by any student characteristics except for weight loss behaviors.

**Conclusion:**

Isolated changes to the school food environment may have unintended consequences unless policymakers incorporate other initiatives designed to discourage overall soda consumption.

## Introduction

Sugar-sweetened beverage (SSB) consumption has become one of the primary targets of childhood obesity prevention efforts in the United States (U.S.) [1]. The negative health effects of SSB consumption are well-documented [2], [3], including recent randomized trials that found SSB consumption was associated with higher weight gain [4], [5]. Sodas are the most popular SSB among adolescents in the U.S. [6], [7] and were the biggest source of calories of any food/beverage group among 14–18 year-olds in 2005–06 [8]. Recent evidence suggests that adolescents have gradually been replacing soda with other SSBs, but soda remains the most heavily consumed sweetened beverage [6].

Numerous policy initiatives have been proposed to reduce consumption of soda and other SSBs, including taxes, marketing regulations, limits on portion sizes, and limits on sugar content [9]–[12]. One of the most widespread initiatives is banning the sale of SSBs within schools [13], [14]. Evidence at all grade levels suggests that policies that ban SSBs have succeeded in reducing students' access to SSBs [15]–[18], though policies at the high school level tend to focus exclusively on soda. Nationally, the proportion of high school students who could purchase soda in school declined steadily from 53.6% in 2006–07 to 25.3% in 2010–11, but most high school students could purchase some type of SSB in school in 2010–11 (87.8%) [19].

Policies have achieved their direct goal of reducing SSB access, but there is relatively little evidence that such changes influence students' consumption of soda or overall SSBs. Several studies reported that consumption was unaffected [16], [20]–[22] even if policies reduced in-school access [16]. These studies utilized several independent data sources that sampled students nationwide. In contrast, however, studies in specific states or districts reported that sweetened beverage restrictions succeeded in reducing sweetened beverage intake [23], [24].

These inconsistent findings could be explained by numerous demographic, behavioral, environmental, or policy characteristics. Previous studies have already suggested that the effects of policies on student behavior may be modified by race/ethnicity [21], [22], or whether the policy targets SSBs other than soda [16] and whether it targets all school venues [24], [25]. Additionally, experts have pointed out that students can easily obtain SSBs from other sources (e.g., home, convenience stores) [10], [20], [26] and thus improvements to the school food environment may be less successful among students who have more access outside of school. The impact of school nutrition policies could also depend on whether school-based changes are complemented by policies in other sectors (e.g., soda taxes). Finally, health consciousness could play a role, as a recent study of calorie labels found that this initiative was more effective among consumers who were less health conscious [27].

To enhance school policy effectiveness, policymakers must consider how effects are modified by factors at the individual, local, or state level. The objective of this study was to determine if the association between school vending machine access and soda consumption varies by demographic, environmental, or policy measures in a nationally representative sample of high school students. We further analyzed dietary behaviors outside of school (e.g., fast food consumption) to assess whether students who do not have access to vending machines in school obtain more unhealthy foods/beverages elsewhere.

## Methods

This cross-sectional study linked student data from the National Youth Physical Activity and Nutrition Study (NYPANS), conducted by the Centers for Disease Control and Prevention (CDC) in Spring 2010, with state-level data on soda taxes, restaurant taxes, and laws governing the sale of soda in schools, collected as part of the Bridging the Gap (BTG) research program. The study was approved by the Institutional Review Boards of the University of Illinois at Chicago and the University of Texas Health Science Center at Houston.

### Student sample

The objective of NYPANS was to measure diet, physical activity, and sedentary behaviors, and environmental determinants of such behaviors, in a nationally representative of 9^th^–12^th^ grade students [28]. Students were sampled using a 3-stage cluster sample design; school and student participation was voluntary, and local permission procedures were followed. The school response rate was 82%, the student response rate was 88%, and the overall response rate was 73%. In total, 10887 public school students participated in NYPANS.

For the purpose of our study, students were excluded because they were missing data on vending machine access within school (n = 691), soda consumption (n = 42), fast food consumption (n = 10), lunch source (n = 496), or other variables of interest (n = 209), or if they were unsure if vending machines that sold sweetened beverages were available in school (n = 1194). Students who were excluded did not differ from the study sample with respect to weight status, soda consumption, or gender, but they were more likely to be racial/ethnic minorities (p<0.001), tended to be in lower grade levels (p<0.001), were less likely to have access to vending machines (p<0.001), were more likely to obtain lunch away from home/school (p = 0.02) and reported more fast food intake (p<0.001). The final study sample included 8245 students in 27 states.

### Student-level measures

All data were obtained using a written questionnaire completed in class. The independent variable of interest was whether the school had “a vending machine that students can use to purchase soda or pop, sports drinks, or fruit drinks that are not 100% juice, such as Coke, Gatorade, or Sunny Delight?” Hereafter, we use the term “vending machines” to refer specifically to this type of vending machine.

Our dependent variables of interest were soda consumption, fast food consumption, and whether students obtained their lunch away from home or school on schooldays. We focused only on soda, not other SSBs, because soda has been the primary target of policy changes on the high school level. Soda consumption was modeled as both a continuous measure of the number of servings consumed in the past 7 days (ranging from none to “4 or more per day”) and a binary measure of whether students consumed at least one soda per day in the past 7 days (hereafter referred to as “daily consumption”). The questionnaire explicitly told students to report consumption of diet soda separately; analyses in this study only utilized data on regular soda consumption. Fast food was modeled as a continuous measure of the number of days in which students consumed fast food in the past 7 days. Students were instructed to include consumption of soda and fast food from all sources regardless of time or location.

Analyses for this study also utilized data on race/ethnicity, sex, grade, availability of food at home, and weight loss behaviors. Students' race/ethnicity was categorized as non-Hispanic White (ref), non-Hispanic Black, Hispanic, and non-Hispanic Other. Home food access was categorized based on whether students usually or always had access to fruits/vegetables or unhealthy snacks (“chips, cookies, or cake”) at home; we cross-classified students as having access to fruits/vegetables only (ref), unhealthy snacks only, both, or neither. Weight loss behaviors were measured using a series of 10 questions that asked students if they had tried to lose weight in the past 30 days through various behaviors. For our purpose, we classified 5 of the 10 behaviors as healthy (exercising, consuming less food or fewer high-fat foods, consuming fewer calories, consuming more fruits/vegetables, drinking more water) and the remaining 5 as unhealthy (skipping meals, fasting, smoking, vomiting or taking laxatives, taking diet pills/powders/liquids without a doctor's advice). Note that these classifications reflect the general health of the behaviors, not the behaviors' effectiveness as weight loss methods. Students were then grouped into 3 mutually exclusive categories of weight loss behavior – those trying to lose weight only through healthy behaviors, those trying to lose weight through at least one unhealthy behavior, and those not trying to lose weight.

We considered analyzing whether the association between vending machine access and dietary behaviors varied by weight status (normal weight, overweight, obese) based on height and weight data obtained by CDC staff. However, this analysis was dropped because the quantity of missing height/weight data was relatively large and exploratory analyses suggested that the associations of interest did not vary by weight status.

### State data

The study included analyses of whether the associations of interest varied by state tax rates for soda sold in grocery stores, state tax rates for foods sold in restaurants, or state laws governing the availability of soda in all school venues. Tax rates were compiled from state statutory and administrative law using primary legal research methods [29], [30] and were confirmed by the state [31], [32]. They were modeled as a continuous measure of the difference between soda/restaurant tax rates versus the general food tax rates (i.e., “disfavored” amount) [33]. Laws regarding the availability of soda in high school vending machines, school stores, and cafeterias (a la carte) in the 2009–10 school year were obtained from the Westlaw and Lexis-Nexis legal research databases and verified against secondary sources [34]–[36]. Laws for the 3 venues were combined to create one binary indicator of whether the state prohibited soda in all 3 venues. Of the 27 states that were sampled as part of NYPANS, eight banned soda in high schools in the 2009–10 school year; of these 8, four states implemented the ban starting in 2009–10 and four states implemented the ban prior to 2009–10. We explored categorizing states based on the length of their ban, but judged that the sample sizes were insufficient for such an analysis.

Both the state tax rate data and the state laws governing soda availability in schools were compiled by the Bridging the Gap research program at the University of Illinois at Chicago.

### Statistical analysis

Due to their respective distributions, the 4 dependent variables were analyzed using different types of regression models – a negative binomial model for total servings of soda in the past 7 days, a Poisson model for fast food consumption in the past 7 days, and a logistic model for daily soda consumption and lunch source. All analyses accounted for the complex sampling design in NYPANS and utilized the ‘margin’ command in Stata, Version 12, to calculate the average marginal effects (AME) of interest.

First, we estimated the association between vending machine access and each dependent variable. Students with no access to school vending machines were the referent, and thus the parameters of interest were hypothesized to be positive (e.g., higher soda consumption in students with access to school vending machines). These models controlled for race/ethnicity, sex, grade, home food access, state median household income (obtained from the 2010 Census), and Census region (Northeast, Midwest, South, West).

Subsequently, we added interaction terms between vending machine access and race/ethnicity, sex, grade, home food access, weight loss behaviors, disfavored soda tax (when modeling soda), disfavored restaurant tax (when modeling fast food and lunch source), and state law banning soda in all school venues. Interaction terms for each variable were modeled separately. When modeling interactions with variables that were not in the original model (e.g., soda tax), the main effect of these variables was also added. Weight loss behavior was initially modeled as a 3-category variable, as described earlier, but we combined 2 of the categories – students who were trying to lose weight through unhealthy behaviors and students who were not trying to lose weight – after analyses revealed that the associations of interest were virtually identical in these 2 groups. When modeling interactions with state taxes and state soda bans, Census region was modeled as a 3-category variable (South/Midwest, Northeast, West) due to the limited distributions of region within categories of state taxes/laws. Home food access was removed from models that included state soda taxes because it may mediate any effect of taxes on soda consumption.

One state, Colorado, was excluded from all analyses of lunch source because it had an extraordinarily high proportion of students who obtained lunch away from school or home (42.0%, versus 6.1% in the remaining sample) and had unusually high leverage as a result.

## Results

Table 1 displays characteristics of the study sample overall and by vending machine access. Nearly all categorical measures had similar distributions regardless of vending machine access. The exception was that students with vending machines were more likely to be from the Midwest (30.0%) and less likely to be from the Northeast (11.1%) relative to students without vending machines (15.8% and 24.9%, respectively.) Students without vending machines were less likely to be obese but more likely to be overweight, and they reported slightly more servings of fast food and soda, relative to students with vending machines.

**Table 1:** Descriptive statistics of study sample, National Youth Physical Activity and Nutrition Study.

		Vending machine a	
Variable	Overall	Yes	No
*N*	8245	6467	1909
*Sex (%)*			
Female	49.1	49.1	49.0
*Race/ethnicity (%)*			
Non-Hispanic White	58.7	60.2	52.8
Non-Hispanic Black	14.6	14.3	15.6
Hispanic	18.3	17.0	23.2
Non-Hispanic Other	8.5	8.5	8.3
*Census region*			
South	37.2	37.6	35.7
Northeast	13.9	11.1	24.9
Midwest	27.1	30.0	15.8
West	21.9	21.4	23.6
*Weight status*			
Overweight (%)	18.0	17.6	19.3
Obese (%)	18.9	19.9	15.3
BMI percentile (mean)	64.2	64.5	63.2
*Dietary behaviors*			
Servings of soda/week (mean)	5.3	5.3	5.6
Days of fast food/week (mean)	1.9	1.8	2.0
*Weight loss behavior (%)*			
Not trying to lose weight	29.2	29.0	30.0
Healthy weight loss behaviors	43.2	43.3	42.7
Unhealthy weight loss behaviors	27.6	27.7	27.3
*Home food access (%)*			
Fruits/vegetables	31.9	31.0	35.5
Unhealthy snacks	13.1	13.0	13.5
Both	37.3	38.5	32.6
Neither	17.7	17.6	18.4
*Where students obtain lunch (%)*			
None	7.0	6.7	8.4
Home	20.0	20.4	18.5
School	67.0	67.3	65.8
Elsewhere	6.0	5.6	7.2

a School vending machine that sells “soda or pop, sports drinks, or fruit drinks that are not 100% juice, such as Coke, Gatorade, or Sunny Delight”

The adjusted associations between vending machine access and soda consumption, fast food consumption, and lunch source are displayed in Table 2. The negative associations indicate that, contrary to the hypothesis, overall soda and fast food consumption were lower among students who had access to vending machines in school. Most notably, students were less likely to report consuming at least 1 soda per day if they had access to school vending machines (23.9%) compared to those who did not have access (27.9%). Students also reported eating fast food on fewer days per week, on average, if they had access to school vending machines (1.82 versus 2.06, AME = -0.24, 95% CI: -0.44, -0.05). They were slightly less likely to obtain a lunch away from home or school, but the difference was more modest.

**Table 2:** Adjusted measures of student soda/fast food consumption and lunch source, by school vending machine access a.

	Vending machine access in school				
	Yes	No	AME b	95% CI	p
Soda servings per week (mean)	5.27	5.80	-0.53	-1.17, 0.11	0.11
Daily soda consumption (%)	23.9	27.9	-4.02	-7.28, -0.76	0.02
Days of fast food per week (mean)	1.82	2.06	-0.24	-0.44, -0.05	0.01
Lunch obtained away from home/school (%)	5.15	7.19	-2.03	-4.59, 0.52	0.12

a Adjusted for race/ethnicity, age, sex, state median income, Census region, and home food access.

b AME  =  Average marginal effect; represents average difference associated with the presence of vending machines that sell sugar-sweetened beverages in school.

**Figure 1:**
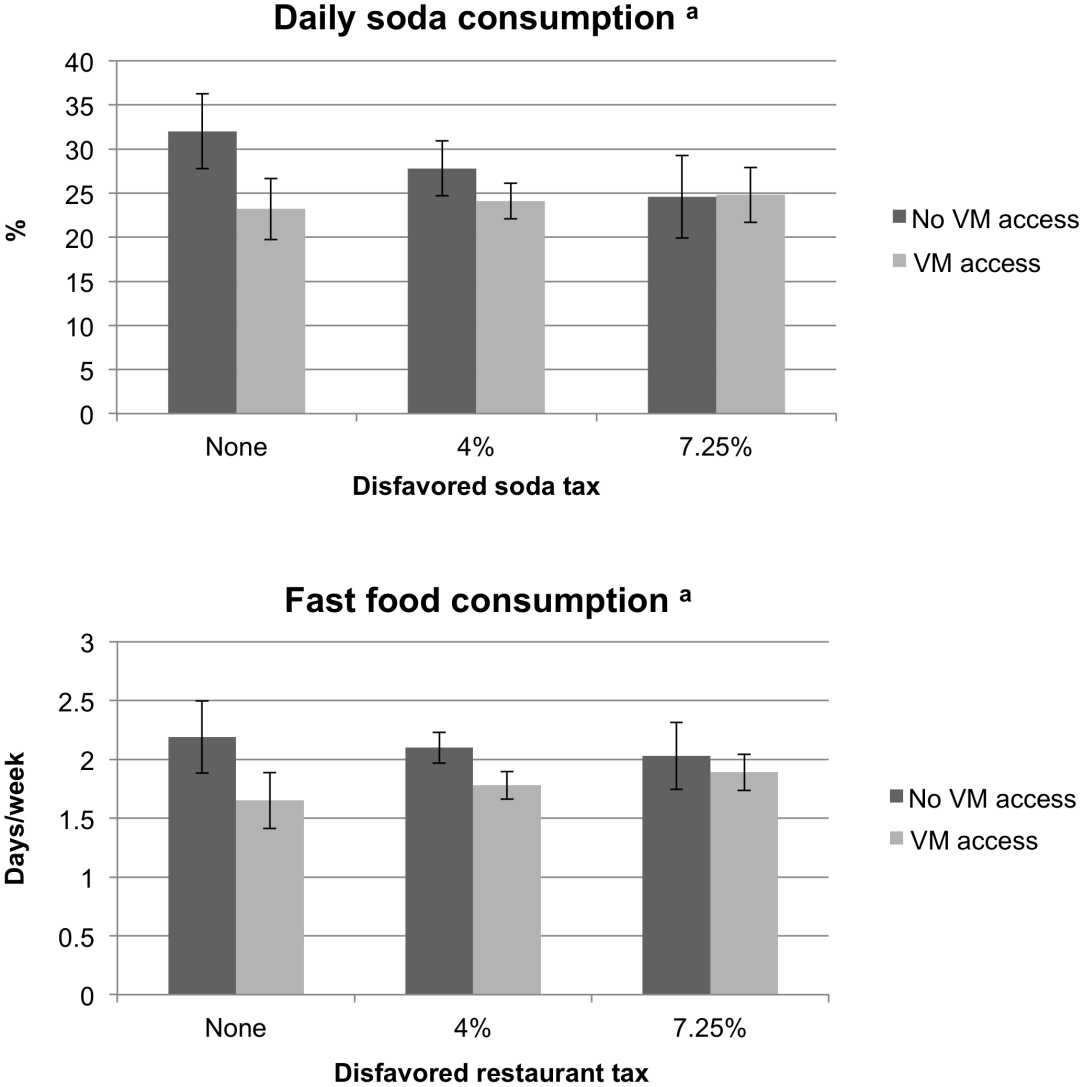
Prevalence of daily soda consumption and mean fast food consumption, by in-school vending machine (VM) access and state taxes. ^a^ As predicted by logistic and poisson models, respectively, adjusted for race/ethnicity, sex, grade, state median income, and Census region.

The associations represented in Table 2 were consistently modified by state tax rates and, to a lesser degree, state laws regarding soda sales in school (Table 3). Results suggested that the unexpected negative associations in Table 2 were generally limited to students in states with no disfavored taxes – i.e., states that taxed soda and restaurant food at the same rate as other foods. For example, in states with no disfavored soda tax, vending machine access was associated with 1.14 fewer servings of soda per week (95% CI: -1.92, -0.36), but this difference became progressively smaller as tax rates increased. The implications of this are displayed in Figure 1. In short, model estimates predict that the inverse association between vending machine access and soda consumption is eliminated if the state tax rate for soda exceeded the general food tax rate by 7.25% (the maximum amount in the study sample.) Similar patterns were observed across all four outcomes.

**Table 3:** Differences in dietary behaviors associated with in-school vending machine access and state initiatives targeting soda.

		Main effects					
		Vending machine access in school		Soda tax, restaurant tax, or in-school soda ban		Interaction	
State measure	Dietary measure	AME a	95% CI	AME a	95% CI	AME a	95% CI
*Soda grocery tax* b							
	Soda servings/week	-1.14	-1.92, -0.36	-0.13	-0.28, 0.02	0.18	0.01, 0.36
	Daily soda consumption c	-8.27	-12.0, -4.50	-0.94	-1.81, -0.08	1.17	0.38, 1.95
*Restaurant tax* b							
	Days of fast food/week	-0.53	-0.88, -0.18	-0.02	-0.09, 0.04	0.05	-0.02, 0.13
	Lunch outside school/home^c^	-7.47	-11.6, -3.37	-0.84	-1.61, -0.08	1.07	0.35, 1.79
*In-school soda ban* d							
	Soda servings/week	-0.64	-1.36, 0.07	0.02	-1.15, 1.19	0.35	-0.82, 1.53
	Daily soda consumption c	-6.23	-9.52, -2.93	-2.40	-7.93, 3.12	5.79	0.00, 11.6
	Days of fast food/week	-0.37	-0.56, -0.19	-0.19	-0.48, 0.09	0.32	-0.02, 0.66
	Lunch outside school/home c	-3.06	-5.71, -0.41	-2.10	-6.89, 2.69	3.22	-1.22, 7.65

a AME  =  Average marginal effect, adjusted for race/ethnicity, sex, grade, state median income, and Census region (models of in-school soda ban also adjusted for home food access.) AME represents the average difference in outcome of interest associated with presence of vending machines that sell sugar-sweetened beverages in school, the state measure of interest (soda tax, restaurant tax, or in-school soda ban); and interaction between the two.

b Disfavored tax (i.e., difference in tax rate relative to general tax rate for foods sold in grocery stores).

c Binary measure.

d State laws that ban the sale of soda in vending machines, school stores, and cafeterias (a la carte).

Similar patterns were also observed when modeling the interaction between vending machine access and state laws banning soda in all venues. In the lower portion of Table 3, the “main effect” of vending machine access represents the association between access and the outcomes of interest in states that did not ban soda; for example, the probability of students drinking soda every day was 6.23 percentage points lower if they had access to vending machines (95% CI: -9.52, -2.93), relative to students without access, in states with no ban. However, the interaction between vending machine access and in-school soda ban was of approximately the same magnitude (AME = 5.79, 95% CI: 0.00, 11.6), essentially meaning that the inverse association between vending machine access and daily consumption was eliminated in states that banned soda. The same pattern was observed for fast food consumption and lunch source – i.e., the inverse “main effect” of vending machine access was balanced by the interaction with state soda ban – though parameter estimates were not as precise for these outcomes.

In contrast to Table 3, the associations between vending machine access and the dependent variables did not consistently vary by most student or environmental characteristics (see Appendix S1). The exception to this was student weight loss behaviors. There was some evidence that the inverse associations noted in Table 2 were limited to students who were not actively trying to lose weight through healthy behaviors. For example, having access to vending machines was associated with 1.14 fewer servings of soda per week if students were not trying to lose weight (95% CI: -2.06, -0.22), but 0.18 more servings per week if students were trying to lose weight (95% CI: -0.55, 0.92).

## Discussion

The effectiveness of removing soda and other SSBs from schools has been questioned by some [10], [20], [26] and advocated by others [14], [37], [38], in part because previous research on policies and in-school SSB access has produced a mixed bag of results [15]–[18], [20]–[24]. Our objective was to explore explanations for the discrepancies by identifying student, environmental, or policy measures that modified the association between vending machine access and overall consumption of soda, fast food, and lunch source. We found evidence that these associations were modified by three variables that each has important policy implications.

Two of these variables were other state policies related to soda consumption – taxes and laws that regulate the sale of soda in school. The unexpected inverse association between vending machine access and soda/fast food consumption was generally limited to states that did not tax soda and restaurant foods, respectively; likewise, the inverse association between vending machine access and daily consumption was limited to states that did not ban the sale of soda in school venues. These results underscore the limitations of isolated changes to the school food environment. In the absence of any other change, children have numerous sources of soda and other high-fat, high-calorie foods and beverages in most middle- and high-income countries [39]. It is unrealistic to expect a single target to have a positive impact within the obesogenic environment to which children are continuously exposed. This challenge is not unique to school nutrition policies or the U.S.; in any country and any domain, health-related or otherwise, policy resistance often occurs when policies singularly focus on specific targets without considering the broader context [40]. Unintended consequences often occur, and our results are an example of individuals possibly overcompensating for an isolated policy/environmental change and engaging in more unhealthy behavior as a result.

This study focused on only one aspect of the school food environment without considering other changes (e.g., strengthening school meal standards). This was done intentionally because our objective was to identify variables that may explain why some policy initiatives have been less successful than other. At a glance, the results may appear discouraging to those who advocate for school nutrition reform, but results should be interpreted in the context of existing literature on school nutrition policies. Studies that evaluated more comprehensive policy changes (e.g., those that required specific limits on nutrition content of competitive foods and beverages) found stronger evidence [24], [25], [41]. The importance of comprehensive change was exemplified by evaluations of school nutrition policy changes in Texas, where minor changes to the school food environment initially had little effect on diet because students compensated [42], but more comprehensive changes later had a positive effect [24]. Furthermore, our study did not analyze how long state policies had been in place, which has been shown to influence the impact of school-based nutrition policies [25].

We also found evidence of unintended consequences among students who were not trying to lose weight. Students who were not engaging in healthy weight loss behaviors consumed considerably more soda and fast food if they did not have access to vending machines at school. Students' healthy weight loss behaviors, in themselves, were a strong, consistent predictor of soda and fast food consumption in this study, but this association became even stronger when vending machines were not available. This raises questions of whether school environmental changes can induce behavioral change among individuals who are not otherwise motivated to lose weight. With that said, this study did not examine whether environmental changes increased students' motivation to lose weight, and this question should be explored in future research.

Future research should examine the mechanisms through which soda or fast food consumption may increase when school vending machines are not available. It is impossible to ascertain from this study whether elevated intake of soda and fast food was a direct consequence of not having vending machines. One could speculate that students who desire soda and do not have access at school simply leave campus to obtain soda elsewhere. Not only would this overcome the absence of vending machines, but it would also lead students to rely on sources that are available 7 days per week (e.g., convenience stores), which could explain why daily consumption was higher when vending machines were not available. If that were the case, it would have important implications for policies that limit students from leaving campus. Another possibility is that schools with vending machines sold other SSBs in place of soda, a trend that has been observed throughout the U.S. [19], and this led students to consume less soda by replacing it with other SSBs. These are only two possible mechanisms, and longitudinal research is needed to study this topic in greater detail.

Results should be interpreted cautiously due to other study limitations. Students were not asked which specific beverages were being sold in vending machines, so it is unknown if those with access could even purchase soda. This is a crucial distinction that should be addressed in future studies. We did not analyze intake of other SSBs because it was beyond the scope of this study, but this is a critical topic given that adolescents' SSB choices have shifted in the U.S. in recent years [6]. We did not analyze weight status either because this association may be confounded by many factors, including the possibility that schools are more likely to remove vending machines if obesity is highly prevalent. The cross-sectional design limited our ability to control for such characteristics. Associations may have been biased by unmeasured variables such as socioeconomic status (SES), a potentially important covariate given the socioeconomic disparities in obesity [43] and the food environment outside of school [26], [44], [45]. Finally, all student data were self-reported and may be vulnerable to measurement error [46], nor did they contain specific measures of nutrient intake. The lack of specific nutrient data makes it difficult to assess whether the associations that we observed were of a sufficient magnitude to have a public health impact. For example, a difference of 0.24 days of fast food, as presented in Table 2, may or may not have an important effect on students' health, depending on the composition of students' fast food consumption.

### Conclusions

School nutrition policy research is a rapidly evolving science. As the evidence base continues to grow, it is becoming apparent that policy effects on student intake are partially determined by interactions between policies and the broader environment in which students live. This study provided another example of where small-scale changes did not appear to improve students' diet, particularly in the absence of other policies. Research suggests that comprehensive changes can have a positive effect, but small-scale changes may potentially backfire unless complemented with nutrition education and other initiatives to improve the nutritional quality of other foods and beverages within and outside of school. Policymakers must be cognizant of how minor changes can have unintended consequences, and investigators should continue to study the dynamics between within-school changes and broader policy and environmental measures.

## Supporting Information

Appendix S1 Interaction between vending machine access and student weight loss, gender, race/ethnicity, and home food access. ^a^ AME  =  Average marginal effect; average difference in outcome of interest associated with presence of vending machines that sell sugar-sweetened beverages in school. ^b^ Adjusted for race/ethnicity, sex, grade, state median income, Census region, and home food access.
(DOCX)
