# Supplementary material for: How State Taxes and Policies Targeting Soda Consumption Modify the Association between School Vending Machines and Student Dietary Behaviors: A Cross-Sectional Analysis
Source: PLoS One. 2014 Aug 1;9(8):e98249. doi: 10.1371/journal.pone.0098249 (PMC4118851; doi:10.1371/journal.pone.0098249)
Supplement: Appendix S1 — Interaction between vending machine access and student weight loss, gender, race/ethnicity, and home food access. a AME = Average marginal effect; average difference in outcome of interest associated with presence of vending machines that sell sugar-sweetened beverages in school. b Adjusted for race/ethnicity, sex, grade, state median income, Census region, and home food access. (DOCX) [file pone.0098249.s001.docx]

**Appendix S1**. Interaction between vending machine access and student weight loss, gender, race/ethnicity, and home food access

|  | **Vending machine access in school** | |  |  |  |
| --- | --- | --- | --- | --- | --- |
|  | **Yes** | **No** | **AME** ^a,b^ | **95% CI** | **p** |
| **Soda servings per week (mean)** |  |  |  |  |  |
| *Healthy weight loss behavior* |  |  |  |  |  |
| No | 5.80 | 6.94 | -1.14 | -2.06, -0.22 | - |
| Yes | 4.42 | 4.24 | 0.18 | -0.55, 0.92 | 0.04 |
| *Gender* |  |  |  |  |  |
| No | 6.08 | 6.43 | -0.35 | -1.21, 0.51 | - |
| Yes | 4.44 | 5.10 | -0.66 | -1.67, 0.34 | 0.52 |
| *Race/ethnicity* |  |  |  |  |  |
| Non-Hispanic White | 5.06 | 5.18 | -0.12 | -0.87, 0.63 | - |
| Non-Hispanic Black | 6.45 | 7.09 | -0.64 | -2.73, 1.45 | 0.69 |
| Hispanic | 5.25 | 6.97 | -1.72 | -3.33, -0.12 | 0.06 |
| Non-Hispanic Other | 4.25 | 4.43 | -0.18 | -2.09, 1.73 | 0.94 |
| *Home food access* |  |  |  |  |  |
| Fruits/vegetables only | 3.36 | 3.78 | -0.42 | -1.11, 0.27 | - |
| Cookies, candy, cake only | 8.70 | 8.83 | -0.13 | -2.03, 1.76 | 0.49 |
| Both | 5.39 | 6.07 | -0.68 | -1.75, 0.39 | 0.99 |
| Neither | 5.76 | 6.21 | -0.45 | -1.67, 0.77 | 0.73 |
| **Daily soda consumption (%)** |  |  |  |  |  |
| *Healthy weight loss behavior* |  |  |  |  |  |
| No | 25.9 | 33.3 | -13.2 | -21.2, -5.25 | - |
| Yes | 20.6 | 20.1 | -5.27 | -7.99, -2.54 | 0.11 |
| *Gender* |  |  |  |  |  |
| No | 27.5 | 32.1 | -4.64 | -9.58, 0.30 | - |
| Yes | 20.1 | 23.5 | -3.36 | -8.24, 1.51 | 0.89 |
| *Race/ethnicity* |  |  |  |  |  |
| Non-Hispanic White | 24.1 | 27.2 | -3.06 | -7.03, 0.90 | - |
| Non-Hispanic Black | 26.8 | 33.1 | -6.30 | -18.6, 5.96 | 0.67 |
| Hispanic | 23.5 | 30.6 | -7.11 | -14.5, 0.29 | 0.33 |
| Non-Hispanic Other | 16.9 | 14.4 | 2.49 | -9.54, 14.5 | 0.45 |
| *Home food access* |  |  |  |  |  |
| Fruits/vegetables only | 14.1 | 17.3 | -3.16 | -7.21, 0.89 | - |
| Cookies, candy, cake only | 39.8 | 41.8 | -1.98 | -9.37, 5.41 | 0.48 |
| Both | 26.3 | 29.1 | -2.82 | -9.16, 3.53 | 0.58 |
| Neither | 23.9 | 33.1 | -9.15 | -16.7, -1.58 | 0.38 |
| **Days of fast food per week (mean)** |  |  |  |  |  |
| *Healthy weight loss behavior* |  |  |  |  |  |
| No | 1.92 | 2.37 | -0.76 | -1.02, -0.49 | - |
| Yes | 1.67 | 1.62 | -0.25 | -0.40, -0.10 | 0.01 |
| *Gender* |  |  |  |  |  |
| No | 1.90 | 2.09 | -0.19 | -0.42, 0.05 | - |
| Yes | 1.73 | 2.05 | -0.31 | -0.52, -0.10 | 0.27 |
| *Race/ethnicity* |  |  |  |  |  |
| Non-Hispanic White | 1.68 | 2.00 | -0.32 | -0.62, -0.02 | - |
| Non-Hispanic Black | 2.30 | 2.55 | -0.25 | -0.70, 0.20 | 0.52 |
| Hispanic | 1.87 | 2.07 | -0.21 | -0.40, -0.01 | 0.47 |
| Non-Hispanic Other | 1.75 | 1.69 | 0.05 | -0.53, 0.64 | 0.26 |
| *Home food access* |  |  |  |  |  |
| Fruits/vegetables only | 1.37 | 1.43 | -0.05 | -0.29, 0.19 | - |
| Cookies, candy, cake only | 2.47 | 2.67 | -0.20 | -0.54, 0.14 | 0.74 |
| Both | 1.93 | 2.27 | -0.34 | -0.67, 0.00 | 0.11 |
| Neither | 1.87 | 2.33 | -0.46 | -0.67, -0.24 | 0.05 |
| **Lunch outside school/home (%)** |  |  |  |  |  |
| *Healthy weight loss behavior* |  |  |  |  |  |
| No | 5.10 | 7.76 | -2.66 | -5.33, 0.00 | - |
| Yes | 5.05 | 6.26 | -1.21 | -4.98, 2.55 | 0.46 |
| *Gender* |  |  |  |  |  |
| No | 6.07 | 6.75 | -0.68 | -3.91, 2.54 | - |
| Yes | 5.08 | 8.20 | -3.12 | -7.08, 0.85 | 0.30 |
| *Race/ethnicity* |  |  |  |  |  |
| Non-Hispanic White | 5.73 | 6.70 | -0.98 | -4.21, 2.26 | - |
| Non-Hispanic Black | 5.81 | 7.84 | -2.02 | -6.63, 2.58 | 0.72 |
| Hispanic | 5.38 | 6.59 | -1.21 | -5.42, 2.99 | 0.91 |
| Non-Hispanic Other | 4.82 | 13.5 | -8.66 | -22.4, 5.05 | 0.17 |
| *Home food access* |  |  |  |  |  |
| Fruits/vegetables only | 4.33 | 5.41 | -1.07 | -4.18, 2.03 | - |
| Cookies, candy, cake only | 6.74 | 7.08 | -0.34 | -5.07, 4.38 | 0.72 |
| Both | 6.93 | 7.16 | -0.22 | -3.52, 3.07 | 0.59 |
| Neither | 4.23 | 11.3 | -7.05 | -14.2, 0.00 | 0.10 |

^a^ AME = Average marginal effect; average difference in outcome of interest associated with presence of vending machines that sell sugar-sweetened beverages in school

^b^ Adjusted for race/ethnicity, sex, grade, state median income, Census region, and home food access
